# Exploring the Impact of Fasting and Fasting-Mimicking Diets on Type 2 Diabetes Management in Adults: A Systematic Review

**DOI:** 10.7759/cureus.70332

**Published:** 2024-09-27

**Authors:** José Amilcar Rivera Regalado, Juan García, Isabella Ramirez, Plinio Hermosilla, Jose Rascon, Sergio José Fausto Girón

**Affiliations:** 1 Microbiology Laboratory of the Faculty of Medicine, Universidad Francisco Marroquín, Guatemala City, GTM; 2 Faculty of Medicine, Universidad Francisco Marroquín, Guatemala City, GTM

**Keywords:** diabetes, fasting diet, fasting-mimicking diet, systematic review, type 2 diabetes

## Abstract

Type 2 diabetes poses significant global health challenges, affecting both the quality of life and healthcare systems. This systematic review evaluates the efficacy of fasting and fasting-mimicking diets (FMD) in managing type 2 diabetes, with a focus on their effects on glycemic control, lipid profiles, and overall metabolic health in adult patients. A comprehensive search of PubMed and Cochrane Library databases identified several studies utilizing various fasting protocols, including intermittent fasting and FMD. Data synthesis and bias assessment were conducted using established methodologies, including the Cochrane Risk of Bias 2 (RoB 2) tool. The review found that fasting interventions significantly improve glycemic control and reduce body weight, with some protocols notably lowering HbA1c levels (p<0.05), highlighting the strong potential of fasting in diabetes management. However, the results varied, suggesting that individual differences in metabolic responses and adherence levels influence outcomes. In conclusion, while fasting and FMD show promise for improving metabolic health and managing diabetes, more standardized research is needed to understand the underlying mechanisms, optimize protocols, and confirm long-term benefits. Future research should prioritize larger sample sizes and extended follow-up periods to inform comprehensive clinical practice guidelines.

## Introduction and background

Dietary interventions play a key role in managing type 2 diabetes, a condition that affects millions worldwide and places a heavy burden on healthcare systems. The number of people with type 2 diabetes is rising quickly, with 10.5% of adults affected in 2021 and predictions suggesting this will increase to one in eight adults by 2045. In 2019, 26.07% of type 2 diabetes-related deaths and 27.08% of disability-adjusted life years (DALYs) were linked to poor dietary choices. The age-adjusted death rate was 4.96 per 100,000 people, and the DALY rate was 232.12 per 100,000​ [1]. Research suggests that specific dietary patterns, such as plant-based, Mediterranean, and low-carbohydrate diets, significantly improve cardiometabolic health in individuals with type 2 diabetes. These diets not only lower HbA1c and triglyceride levels but also offer potential benefits in reducing overall cardiovascular risks and in promoting weight management [2]. The integration of nutritional strategies like the ketogenic diet and intermittent fasting (IF) has shown positive effects on glycemic control and insulin sensitivity, which are crucial for delaying the progression of type 2 diabetes and minimizing the risk of developing related complications [3,4].

Expanding upon these foundational dietary approaches, fasting and fasting-mimicking diets (FMD) have emerged as innovative interventions for enhancing metabolic health. IF, characterized by alternating periods of fasting and eating, has demonstrated significant benefits in enhancing metabolic flexibility, mobilizing fat, and preserving lean body mass [5]. Research has further revealed that IF contributes to reduced insulin levels, improved glucose metabolism, and diminished cardiovascular risk factors [6]. Moreover, fasting is linked to increased insulin sensitivity and reduced low-density lipoprotein (LDL) cholesterol, accompanied by favorable neuroendocrine shifts [7,8]. Although fasting is generally well-tolerated, some individuals may experience mild side effects such as headaches, lethargy, and mood swings. Collectively, these findings underscore the potential of fasting and FMD as effective strategies for type 2 diabetes management, warranting further investigation to determine their long-term efficacy and safety across diverse populations [6-8].

Fasting and FMD provide substantial flexibility and additional benefits beyond traditional dietary interventions for type 2 diabetes. Diverse fasting methods, including IF, time-restricted feeding (TRF), and periodic fasting (PF), have been proven effective in enhancing glycemic control, insulin sensitivity, and body weight management [9]. These methods play a crucial role in reducing insulin resistance and LDL cholesterol, enhancing fat metabolism, and decreasing inflammation. Additionally, IF has demonstrated a positive impact on overall metabolic health by improving glucose metabolism and reducing cardiovascular risk factors [10]. It is essential, however, to implement these fasting regimens under strict medical supervision to prevent adverse effects such as hypoglycemia, especially in insulin-dependent patients [11]. Grajower and Horne highlight the necessity of personalized care, including tailored medication adjustments and rigorous glucose monitoring, to maximize the benefits and minimize the risks associated with fasting in diabetic populations. Despite the encouraging outcomes, the need for more comprehensive long-term studies remains to confirm the enduring benefits and safety of these dietary strategies in diverse diabetic cohorts [11,12].

## Review

Methodology

Question Design

The research question was formulated using the PICOS (patient or population, intervention, comparison, outcomes, and study design) format (Table 1) [13]. The question posed is as follows: How effective are fasting and FMD in improving outcomes for adult patients with type 2 diabetes?

**Table 1:** PICOS framework This table outlines the PICOS framework used to formulate the research question and to guide the search for studies. PICOS: patient or population, intervention, comparison, outcomes, and study design

Population	Intervention	Comparison	Outcomes	Study design
Adults with type 2 diabetes	Fasting and fasting-mimicking diets	Standard diabetic diets without fasting components	Improvement in glycemic control (measured by HbA1c levels), changes in body weight, lipid profiles, insulin sensitivity, and quality of life	Randomized controlled trials, controlled clinical trials, and observational studies

Inclusion and Exclusion Criteria

The population studied included patients diagnosed with diabetes, aged over 18. The interventions encompassed various types of fasting, such as the FMD, IF, alternate-day fasting, water fasting, and calorie restriction. Control groups adhered to standard diets. We included studies where outcomes were linked to favorable therapeutic responses and other surrogate markers, including randomized controlled trials, controlled clinical trials, and observational studies. Additionally, we considered a range of outcomes to provide a comprehensive evaluation of glycemic control, lipid profile, body weight and composition, blood pressure, inflammatory markers, quality of life, adherence and sustainability, incidence of diabetes-related complications, and ketone bodies.

This approach ensures a thorough understanding of how various fasting regimens impact multiple aspects of diabetes management and patient health, which could inform future dietary guidelines and therapeutic strategies for diabetes care. We excluded case reports, reviews, and nonclinical trials. Studies involving animals, pediatric patients, and pregnant participants were also excluded.

Search Strategy

The search strategy was executed using the PICOS format (Table 1), which guided the comprehensive review of all eligible literature [13]. This method was applied to databases such as PubMed and the Cochrane Library, including research papers available up to the date of access on September 6, 2024. Studies were restricted to those published in the English language. No additional limitations or exclusions were set regarding the date of publication, geographical area, or the language of the studies.

Selection of Studies

The search results were initially evaluated by all reviewers, during which duplicates were manually removed. The remaining studies were then meticulously analyzed, starting with the evaluation of titles and abstracts. To facilitate organization and further analysis, the relevant data from these studies were also added to an Excel spreadsheet. This process involved all reviewers working collaboratively. At each stage, the inclusion or exclusion of studies required a consensus among the reviewers to ensure consistency and accuracy. In cases of disagreement, all reviewers presented their points of view, followed by a detailed discussion and a vote, thereby ensuring comprehensive agreement on each step of the article selection and exclusion process.

Data Extraction and Synthesis

A standardized data extraction form was used, and all participants reviewed the data, forming the basis for the reviewers' work. This method ensured the systematic capture of relevant information while minimizing potential data selection biases. In instances of missing or ambiguously described data, the study investigators were contacted for clarification. Any discrepancies in data extraction were resolved through discussions among the reviewers until a consensus was reached.

The extracted data were systematically organized into Excel tables and charts, summarizing the main findings from each study. This arrangement facilitated comparisons and supported thematic analysis, helping to identify themes, patterns, and gaps in both qualitative and quantitative data.

Risk of Bias Assessment

The quality of the four studies included in this review was assessed using the Cochrane Risk of Bias 2 (RoB 2) tool. To minimize bias, each included clinical trial was independently assessed by two reviewers using the RoB 2 Crossover Beta Excel tool, with an emphasis on blinding [14,15]. Discrepancies were resolved through discussion and detailed analysis until a consensus was achieved.

Results

Description of the Studies

**Figure 1:**
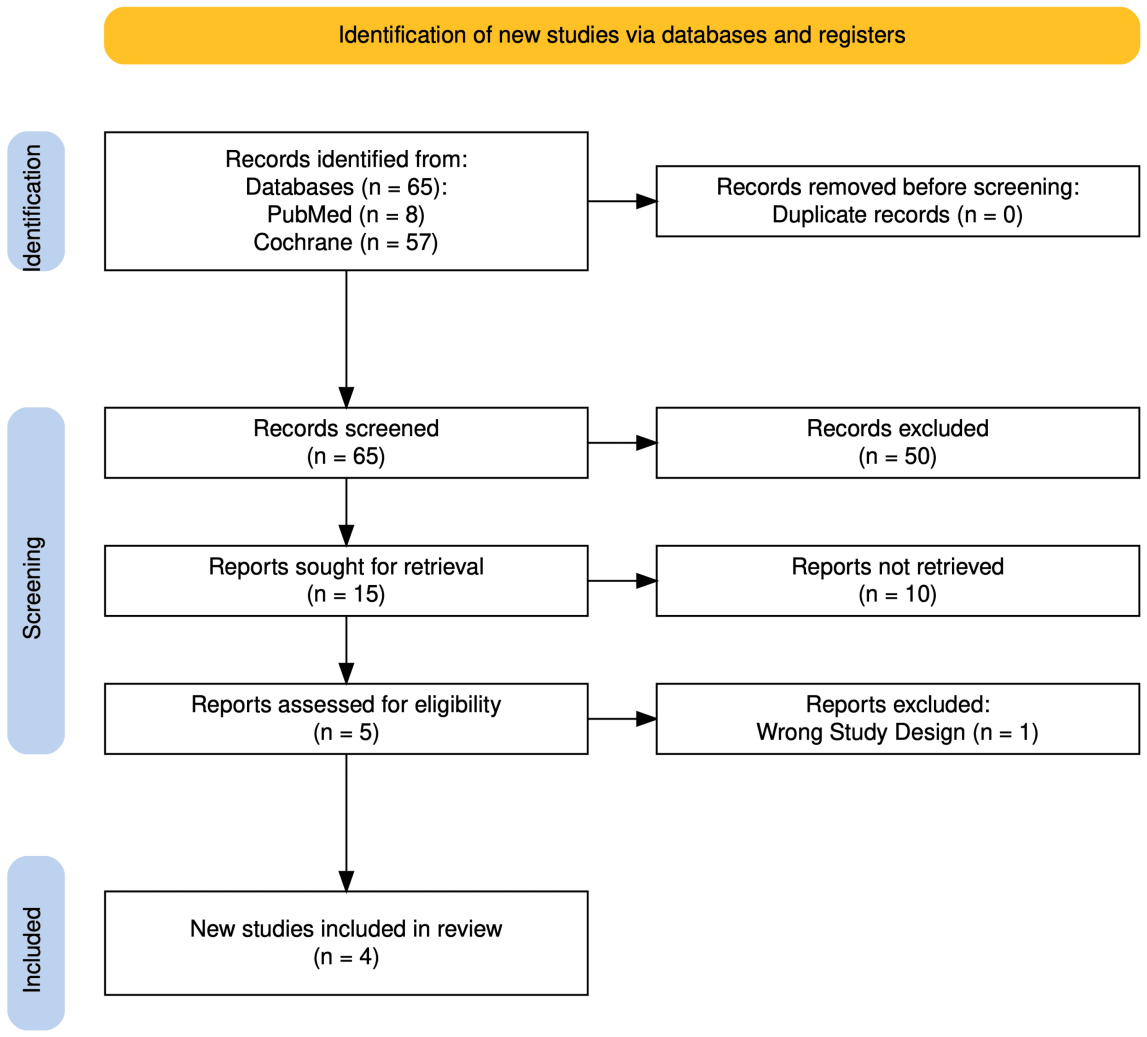
PRISMA flow diagram showing the identification of eligible and participating studies PRISMA: Preferred Reporting Items for Systemic Reviews and Meta-Analyses Reference: [16]

Our systematic search initially identified a total of 65 articles from databases. After careful screening and assessment, four articles met our eligibility criteria (Figure 1) [16]. These studies collectively encompassed data from a significant number of patients, with three of the selected studies being clinical trials and one being an observational study.

Study Characteristics

Table 2 provides an overview of the key characteristics of the studies encompassed in this analysis. These studies were published within the timeframe spanning from 2015 to 2024 and distributed among the following countries: USA (n=2), Thailand (n=1), and Italy (n=1). Sample sizes ranged from seven to 209 participants. The majority of participants were women. The ages of participants ranged from 30 to 75 years. The duration of treatment ranged between four weeks and six months (Table 2).

**Table 2:** Studies' characteristics and key findings IF: intermittent fasting; iTRE: intermittent fasting plus early time-restricted eating; CR: calorie restriction; W: women; M: men; AUC: area under the curve; LDL: low-density lipoproteins; HDL: high-density lipoproteins; ANOVA: analysis of variance; NEFA: nonesterified fatty acids

Authors	Sukkriang and Buranapin [17]	Nuttall et al. [18]	Tricò et al. [19]	Teong et al. [20]
Publication year	2024	2015	2023	2023
Journal	Journal of Diabetes Investigation	Metabolism Clinical and Experimental	Diabetologia	Nature Medicine
Country	Thailand	USA	Italy	USA
Study design	Randomized controlled trial	Randomized crossover study	Randomized, parallel-arm, open-label, controlled trial	Open-label, parallel-group, three-arm randomized controlled trial
Study duration	Three months	Three days per intervention, with a four-week washout period between each arm	12 weeks	Six-month intervention phase followed by a 12-month follow-up
Sample size	108	7	27	209
Population	Obese patients with type 2 diabetes	Male subjects with untreated type 2 diabetes	Individuals with type 2 diabetes and overweight/obesity	Adults at increased risk of developing type 2 diabetes
Age	30-60 years old	45-75 years old	67.2±7.9 years	58±10 years
Co-intervention	Obese diabetic patients were randomized into IF 16:8, IF 14:10, or a control group with a standard diabetic diet. IF groups fasted three days/week. All received dietary and exercise guidance to compare weight loss and metabolic outcomes	Fasting and carbohydrate-free diet	Both groups received diets with matched energy restriction and macronutrient distribution (50% carbohydrate, 30% fat, 20% protein)	Nutritional support was provided to participants in the iTRE and CR arms
Sex	W: 58, M: 41	M: 7	W: 13, M: 14	W: 119, M: 90
Outcomes	The primary outcome was the change in weight loss; secondary outcomes were the change in fasting glucose, HbA1c, and lipid profiles. These were assessed to evaluate the effectiveness of IF on metabolic health	The study evaluated plasma glucose, insulin, and glucagon levels	The primary outcome was the between-group difference in HbA1c at 12 weeks. Secondary outcomes included body composition, glucose monitoring, and metabolic markers	The primary outcome was the change in glucose AUC in response to a mixed-meal tolerance test at month 6 in iTRE versus CR. Secondary outcomes included changes in body weight, body composition, fasting and postprandial markers of glycemia, and cardiovascular and liver health
Main results	IF 16:8 and 14:10 significantly reduced weight and improved metabolic markers in obese type 2 diabetes patients. The IF 16:8 group showed a 4.02% weight loss, while IF 14:10 had a 3.15% reduction. Both groups had decreases in fasting glucose, HbA1c, triglycerides, and LDL, with increases in HDL. Statistical analyses confirmed IF as an effective strategy for improving metabolic health in this population	Both interventions significantly reduced plasma glucose and insulin levels, with fasting showing a greater glucose drop (196 mg/dL to 127 mg/dL). Insulin levels decreased from 18 μU/ml to 14 μU/ml with fasting, while glucagon remained unchanged in both groups. The Friedman test confirmed fasting's stronger impact on glucose and insulin regulation	Both dietary groups had similar reductions in body weight, fat mass, HbA1c, fasting glucose, and other metabolic markers. No significant differences were found in GLP-1, GIP, or NEFA levels. Statistical analyses used Fisher's exact test, Mann-Whitney U test, and two-way ANOVA, with exploratory analysis stratifying participants by carbohydrate intake tertiles	iTRE showed greater improvement in postprandial glucose AUC at six months compared to CR (-10.10 vs. -3.57 mg/dL/min; p=0.03). No significant difference was seen at 18 months (p=0.17). iTRE also had greater reductions in fasting NEFA and postprandial insulin AUC at six months. Both iTRE and CR reduced systolic and diastolic blood pressure versus standard care. iTRE significantly reduced plasma β-hexosaminidase activity, indicating improved liver health

Assessment of Risk of Bias in Individual Studies

**Figure 2:**
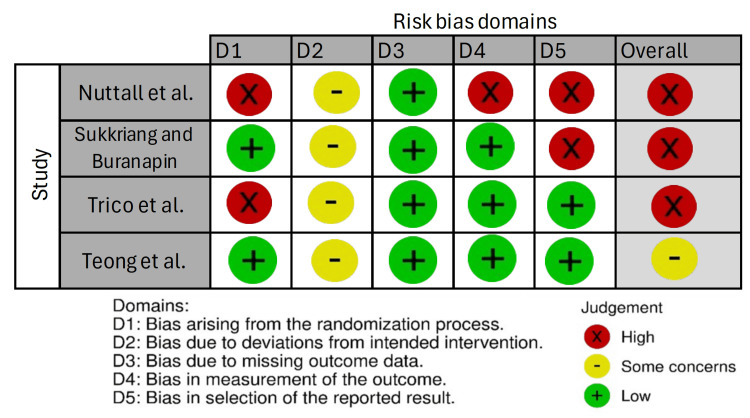
Risk of bias evaluation in included randomized controlled trials using the Cochrane Risk of Bias 2 tool References: [14,15,17-20]

Reviewers performed the quality assessment of included trials using the Cochrane RoB 2 tool. The appraisal of the risk of bias of each included study is summarized in Figure 2.

Discussion

This systematic review synthesized findings from four studies examining the effects of various dietary interventions on metabolic health in populations with or at risk of type 2 diabetes. These interventions, ranging from IF to carbohydrate-free diets and time-restricted eating, aimed to improve key metabolic markers such as glycemic control, lipid profiles, and body weight.

Population-Specific Responses and Comparative Effectiveness of Dietary Interventions

Variations in population characteristics, such as baseline metabolic health, age, and the severity of diabetes symptoms, highlight the need for personalized dietary recommendations. For instance, a study examined older adults with established type 2 diabetes and overweight conditions, finding comparable benefits from both a Mediterranean-style diet and time-restricted eating. This suggests that less restrictive diets can still offer significant health benefits in managing chronic conditions, particularly in older populations. Our review also found that while all dietary interventions were effective to some degree, their impacts varied between studies. For example, some studies reported significant improvements in weight loss and glycemic control from IF and time-restricted eating. However, these benefits were not consistently observed across all studies, given that some focused on acute metabolic changes from short-term dietary interventions, indicating that the duration and type of dietary intervention may play critical roles in their overall effectiveness [17-20].

The Role of Intervention Timing, Methodological Approaches, and Statistical Analysis in Evaluating Dietary Interventions

Significant short-term improvements in postprandial glucose control from early time-restricted eating were found, which were not observed with standard calorie restriction. This finding emphasizes the importance of not only what we eat but also when we eat, particularly in managing metabolic diseases. However, the lack of sustained benefits at the 18-month follow-up raises questions about the long-term effectiveness of such interventions. Additionally, the diverse methodologies employed, ranging from randomized controlled trials to crossover studies, reflect the complexity of nutritional research. Each design has its strengths and limitations, which must be considered when interpreting results. For instance, crossover designs allow for within-subject comparisons, potentially reducing variability due to individual differences in metabolic responses to diets. Advanced statistical methods, such as linear mixed-effects models and likelihood ratio tests, have further provided deeper insights into the data, clarifying the specific conditions under which dietary interventions are most effective. However, the lack of significant differences in some outcomes suggests that additional factors, such as behavioral or genetic influences, may impact the success of these interventions. These methodological and statistical considerations highlight the need for a nuanced approach to both the timing and design of dietary intervention studies [17-20].

Practical Implications, Research Gaps, and Future Directions

Clinicians must weigh both the efficacy and practicality of dietary interventions when making recommendations. While IF and time-restricted eating show promise, it is essential to tailor these approaches to individual patient lifestyles and preferences to enhance adherence and overall effectiveness. Despite encouraging findings, this review highlights several research gaps. There is a pressing need for longer-term studies involving larger and more diverse populations to better understand the effects of dietary interventions on chronic metabolic conditions. Additionally, future research should examine the integration of behavioral support mechanisms to promote adherence, ensuring that dietary interventions achieve sustained, long-term benefits.

## Conclusions

Fasting and FMD offer potential benefits in managing type 2 diabetes, particularly for improving glycemic control and weight loss. However, their effectiveness varies based on individual factors and intervention duration. More long-term, standardized studies with diverse populations are needed. Clinicians should focus on personalized dietary plans to enhance adherence and effectiveness.
